# An almost complete cranium of *Asoriculus gibberodon* (Petényi, 1864) (Mammalia, Soricidae) from the early Pliocene of the Jradzor site, Armenia

**DOI:** 10.1186/s13358-025-00357-6

**Published:** 2025-04-14

**Authors:** Hugo Bert, Loic Costeur, Sergei Lazarev, Georg Schulz, Davit Vasilyan, Olivier Maridet

**Affiliations:** 1 CNRS UMR 5125 “Paléoenvironnements & Paléobiosphère” Université Claude Bernard Lyon 1, Rue Raphaël Dubois 2, 69622 Villeurbanne, France; 2 Ecole Normale Supérieure de Lyon, Parvis René Descartes 15, 69342 Lyon Cedex 07, France; 3 Natural History Museum Basel, Augustinergasse 2, 4001 Basel, Switzerland; 4 Jurassica Museum, Route de Fontenais 21, 2900 Porrentruy, Switzerland; 5 Department of Geosciences, University of Fribourg, Chemin du Musée 6, 1700 Fribourg, Switzerland; 6 Department of Biological Engineering Biomaterials Science Center, University of Basel, Hegenheimermattweg 167C, 4123 Allschwil, Switzerland

**Keywords:** Soricinae, Dental morphology, Cranial anatomy, Inner ear, Pliocene, Armenia

## Abstract

We describe an almost complete fossil cranium of a shrew, identified as *Asoriculus gibberodon* (Petényi, 1864) from the early Pliocene of Jradzor site, Armenia. The sedimentary unit, which yielded the specimen, is an 11-m-thick package composed of white thinly-parallel-laminated diatomite laying at the base of the Jradzor section. It was dated at 4.29 ± 0.09 Ma based on the magnetostratigraphy and ^40^Ar/^39^Ar radioisotopic dating of a tephra layer located at the top of the diatomite package. The skull from Jradzor shows several synapomorphies that allow its assignment to the Soricinae subfamily and Neomyini tribe. Among Neomyini, as far as the cranium anatomy is known, the specimen from Jradzor is most similar to that of *Soriculus* and *Episoriculus*. Both petrosal bones are preserved and are studied thanks to a 3D modelling of their morphology based on a CT-scan. Compared with other eulipotyphlans, the bony labyrinth of *A. gibberodon* from Jradzor shows a morphology typical of soricids. Its anatomy also indicates a high-frequency auditory capability similar to that of modern shrews but cannot confirm an echolocation system neither does it shows any feature that can be related to a specific locomotory adaption or ecological characteristic. The discovery of this cranium inside diatomites, corresponding to a distal lacustrine environment, raises the question of the possible semi-aquatic adaptation of this species (this adaptation being known for other extant species of the family). However, *Soriculus* and *Episoriculus*, the two genera closest to *Asoriculus* based on cranial anatomy are not semi-aquatic and are clearly distinguished from semi-aquatic *Neomys* shrews. The inner ear morphology is more similar to that of terrestrial shrews despite the general similarities among soricids and suggests an echolocation-based orientation using high frequencies to navigate through low vegetation, which is often essential in high metabolic rate organisms to reduce energy expenditure. We therefore propose a terrestrial locomotion for *A. gibberodon*, consistent with its previously proposed paleoecological model, depicting it was a terrestrial species inhabiting wet or humid environments in close proximity to permanent bodies of water.

**Supplementary Information:**

The online version contains supplementary material available at 10.1186/s13358-025-00357-6.

## Introduction

The Soricidae are the fourth most species-diverse mammal family formed by more than 380 extant species of shrews with a large geographic distribution covering Africa, Europe, and Asia, as well as parts of America (Wilson and Reeder, 2005). These animals are 2 g to 100 g tiny mouse-like predators with long pointed snouts. They are mostly terrestrial, although some species are semi-aquatic or arboreal (Hutterer, 1985). Some of the largest species of shrews (e.g., *Blarinella brevicoda* Say, 1823; *Neomys fodiens* Pennant, 1771 and *N. milleri* Mottaz, 1907) are among the rare present-day venomous mammals. Their salivary paralysing venom allows them to hunt relatively large-size prey (Furió et al., 2010; Kowalski & Rychlik, 2021). Terrestrial shrews are also able to orient themselves using an echo-based mechanism when foraging (Catania et al., 2008; Gould et al., 1964).

The Soricidae are divided into two present-day subfamilies with great biological distinctions: the red-toothed Soricinae having a constant high metabolic rate and the white-toothed Crocidurinae, which, in contrast, are able to enter into a state of torpor (Taylor, 1998). Additionally, a third fossil subfamily is also currently accepted, named either Crocidosoricinae or Myosoricinae (see Furió et al, 2007 and Hutterer, 2005a respectively).

Despite their diversity and ecology, soricids are often neglected in palaeontological studies mostly because of the rare preservation of their cranial remains and their slow evolutionary rate (Reumer, 1984) inducing little value for biostratigraphic purposes. Moreover, the great majority of fossil shrews are represented by isolated teeth and mandibles, and the very thin post-palatal parts of the skull are hardly ever preserved (Repenning, 1967). Likewise, the inner ears of shrews are poorly known because they have been seldom studied on extant species and have never been previously described in the fossil record.

In the present study, we describe an almost complete fossil cranium of a shrew, identified as *Asoriculus gibberodon* (Petényi, 1864) from the early Pliocene diatomite deposits of Jradzor site, Armenia (Lazarev et al., 2023). This specimen allows us to increase our knowledge of the cranial anatomy of *A. gibberodon* and to describe its so far unknown inner ear morphology.

## Geological context

### Jradzor locality

**Fig. 1:**
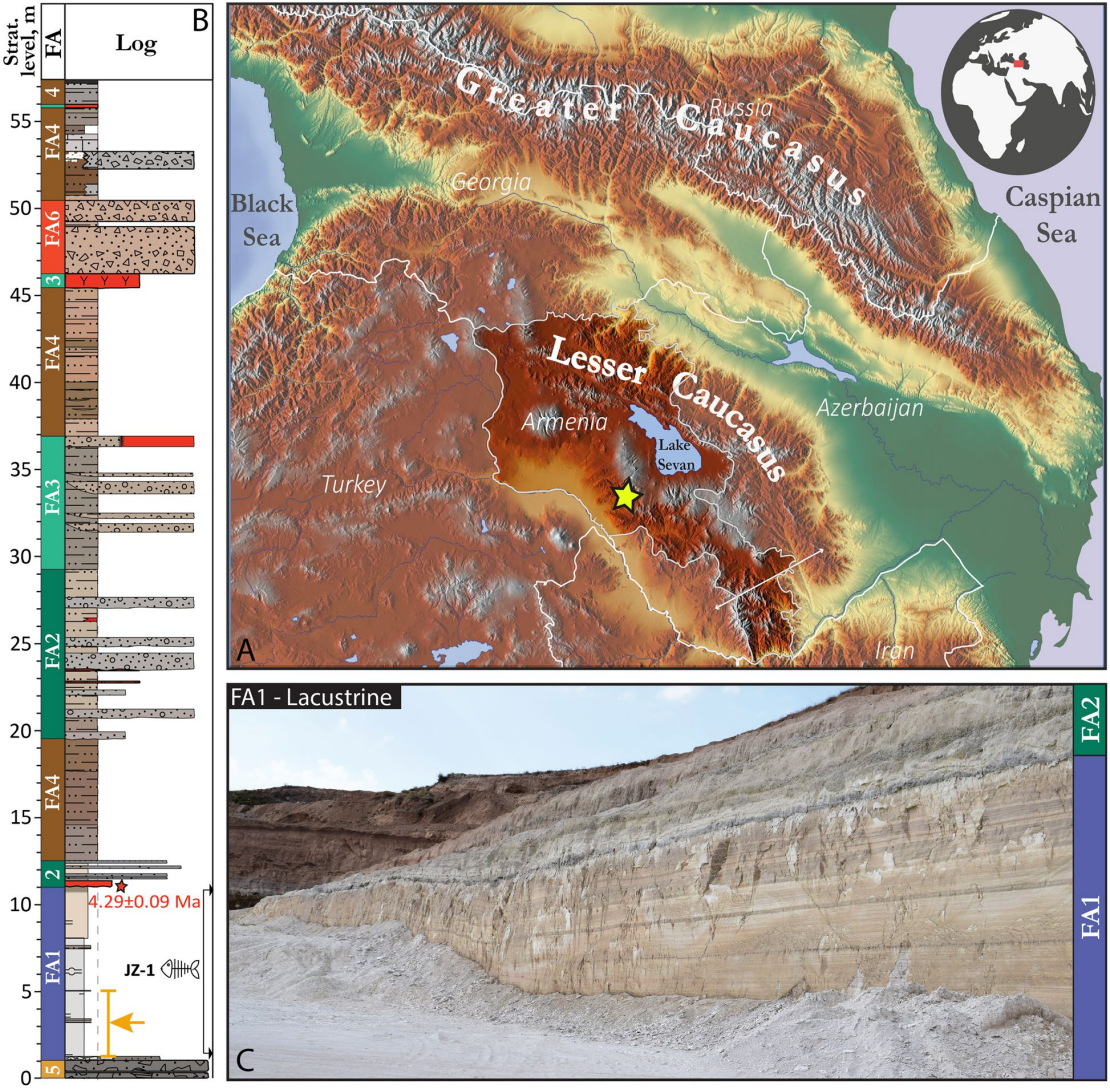
Geographic location of Jradzor site (**A**) accompanied by the log of the section where the stratigraphic position of the fossil find is indicated by orange arrow (**B**). An overview photo of the diatomite package (**C**)

The specimen of the shrew studied herein was excavated from the Jradzor diatomite mine (40°1′42.53ʺN 44°48′53.92ʺE) near the village of Narek (Fig. 1A). The region is located within the Plio-Pleistocene volcanic province of Gegham, in the southern part of the Armenian Lesser Caucasus (Lazarev et al., 2023).

The sedimentary unit (Facies association 1 in Lazarev et al., 2023), which yielded the specimen, lays at the base of the Jradzor section. It is an 11-m-thick package composed of white thinly-parallel-laminated diatomite in its upper part also containing layers of tuffaceous sandstones and tephras (Fig. 1B, C). In addition to the soricid skull studied in this paper, this unit has yielded fossils of fishes, frogs, a turtle, a lagomorph, and aquatic plants (Lazarev et al., 2023). Paleoenvironmental reconstructions suggest that the diatomites were deposited in a short-lived dammed lake that later became a subject for pyroclastic density currents. The herein-studied soricid was found on the lower half of the diatomite (between 1.3 and 5 m), corresponding to a stable, relatively deep-water setting (Fig. 1C). General pyritization of the fish remains found in the same interval and accumulation in a stable water column suggest poorly oxygenated environments, which probably permitted the exceptional preservation of the material (Lazarev et al., 2023).

The diatomite package was dated at 4.29 ± 0.09 Ma by Lazarev et al. (2023) based on the magnetostratigraphy and ^40^Ar/^39^Ar radioisotopic dating of a tephra layer located at the top of the diatomite package. This age correlates to the MN15 in the European biozonation framework (Raffi et al., 2020). The correlation can be supported by the presence of fossiliferous horizons from the 10-m overlying layers with small mammalian fauna of the same MN15 zone.

## Material and methods

### Classification, terminology and measurements

**Fig. 2:**
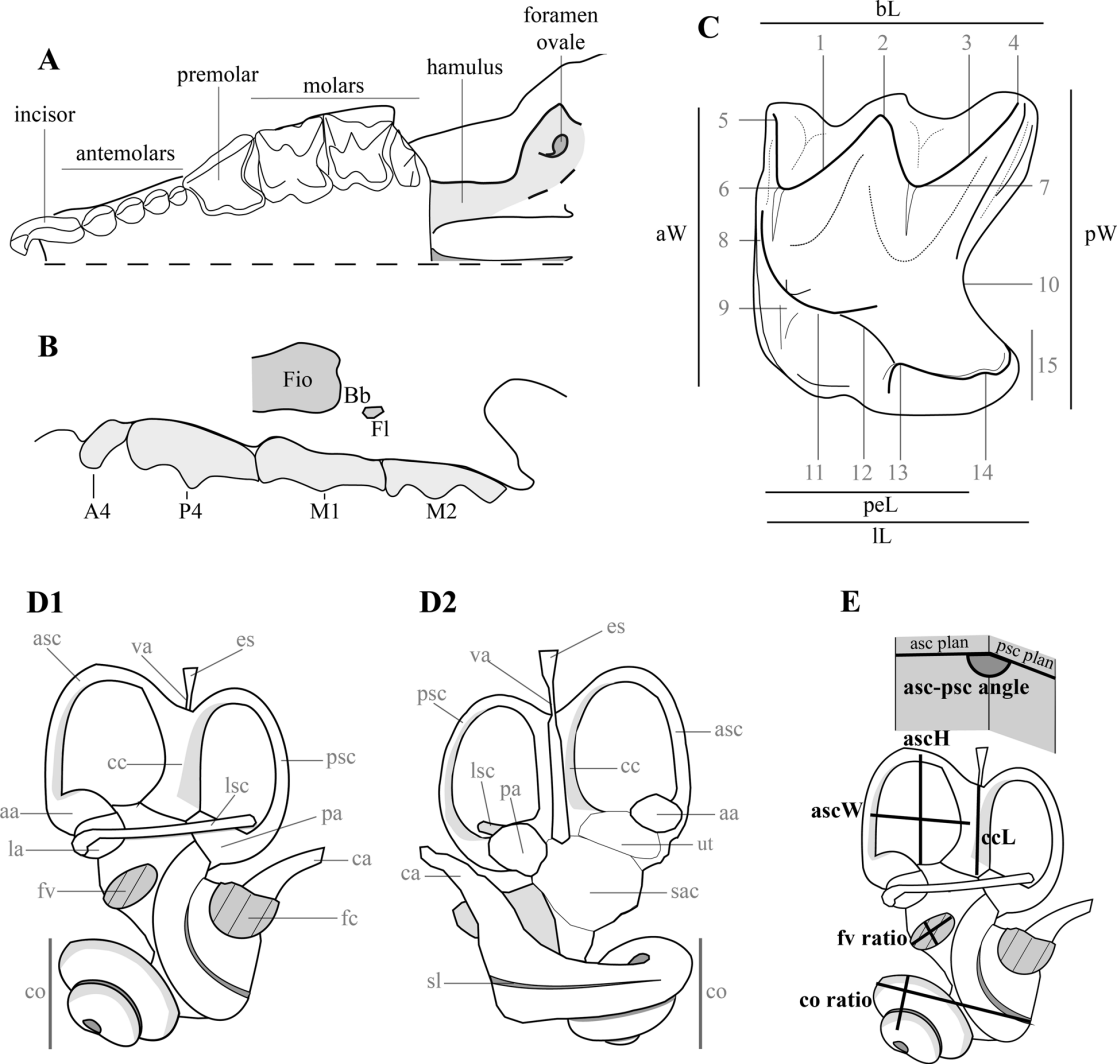
Terminology and measurements; **A** left half of the ante-glenoid part of a soricid cranium in occlusal view; **B** left buccal view of a maxilary of a soricid cranium: **Fio**: infraorbital foramen; **Fl**: lachrymal foramen; **Bb**: bony bridge; **C** molar nomenclature and measurements following Osborn (1907) applied to shrews in Reumer (1984). **1** postparacrista, **2** mesostyle, **3** postmetacrista, **4** metastyle, **5** parastyle, **6** paracone, **7** metacone, **8** paraloph (= anteprotocrista if not reaching the paracone), **9** protocone, **10** posterior emargination, **11** postprotocrista (= metaloph if reaching the metacone), **12** hypoloph, **13** hypocone, **14** hypoconule, **15** hypoconal flange; **D**, **E** inner ear nomenclature and measurements following Costeur (2014) (**D1**, **E** posterolateral view, **D2** anteromedial view). See text for abbreviations

The classification used in this paper follows Reumer (1998a). Herein, the Neomyini tribe is used as equivalent to the extent Nectogalini tribe. In this study, all teeth between the first incisor and the fourth premolar are named antemolars (Fig. 2A, B). The terminology and measurements of teeth follow those proposed by Reumer (1984), which itself is modified after the mammal tooth terminology of Osborn (1907) (Fig. 2C). Measurements were taken using the Amira® software. All measurements are given in millimetres with a precision of 0.01 mm.

The terminology and orientation of inner ears bony labyrinths follow Costeur (2014) modified from Ekdale (2013) for measurements, and from Orliac et al. (2012) and Schwarz (2012) for terminology and orientations (Fig. 2D–Dc). In our study, inner ears are always illustrated with two of the three semi-circular canals aligned in the horizontal and vertical planes to simplify the comparison. An exception has been made for the right inner ear of the Jradzor specimen shrew because its semi-circular canals have been deformed by post-mortem mechanical deformation.

### Referred specimen

The studied specimen from Jradzor is stored in the Institute of Geological Sciences, National Academy of Sciences of the Republic of Armenia, Yerevan. It is catalogued under the number IGS JRD-19/100.

**Fig. 3:**
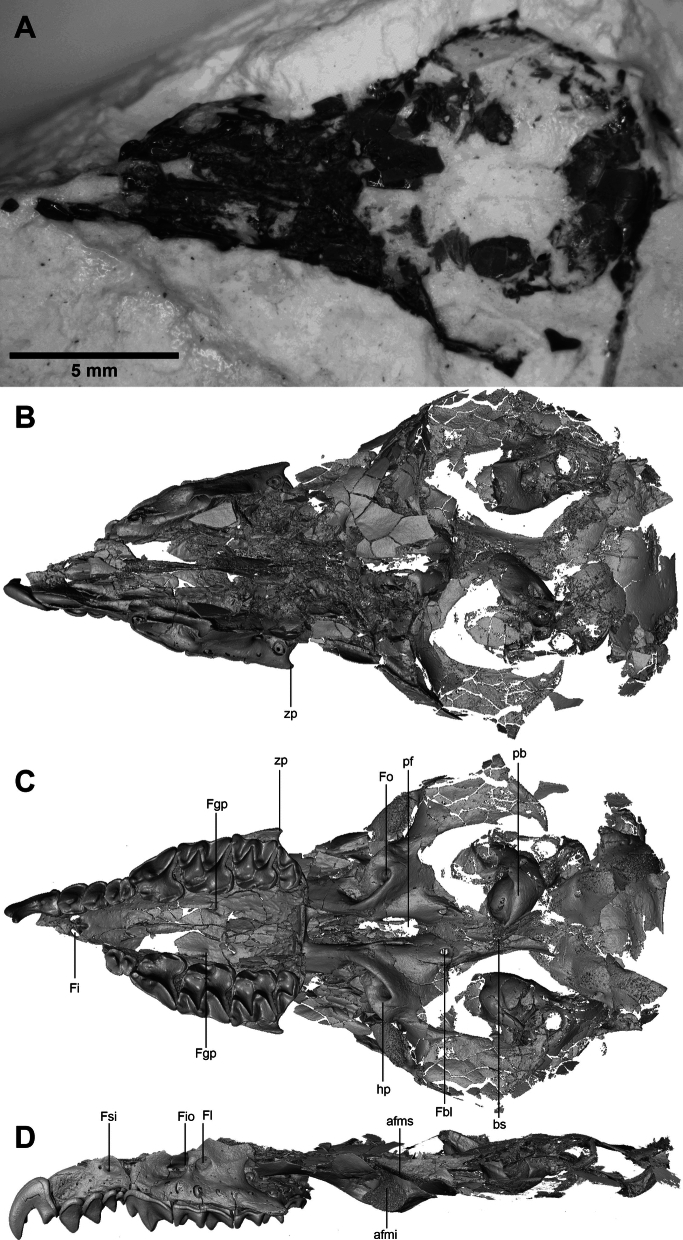
The Jradzor soricid cranium. **A** Photo of the fossil skull (dorsal view) partly embedded in the diatomite sediment; **B**–**D** 3D model of the same skull extracted from the CT-data: **B** dorsal view, **C** ventral view, **D** left lateral view. **afmi**: lower articular facet of the mandible; **afms**: upper articular facet of the mandible; **bs**: basioccipital suture; **Fbl**: foramen basisphenoidalis lateralis; **Fgp**: greater palatine foramen; **Fi**: incisive foramen; **Fio**: infraorbital foramen; **Fl**: lachrymal foramen; **Fo**: foramen ovale; **Fsi**: foramen supra infraorbitalis; **hp**: hamulus of pterygoid; **pb**: petrosal bone; **pf**: pterygoid fossa; **zp**: zygomatic processes

The specimen is an almost complete cranium, partially flattened (Fig. 3). All upper teeth are present except the incisor and the first two antemolars of the right maxillary. Two bony labyrinths of the inner ear are preserved. The specimen is probably a young adult as indicated by the very little wear on the tips of teeth (Hutterer, 2005b; Vesmanis & Vesmanis, 1979). It is not possible to have information about its gender because there is no sexual dimorphism in shrews (Zidarova, 2015).

### 3D reconstruction

The cranium from Jradzor is partly embedded in the diatomite, so the ventral view is not visible (Fig. 3A). Since the specimen is very fragile, it was not fully extracted from the sediment but studied using computed tomography (CT). µCT data were obtained with the CT-scanner Nikon XT H 320 at the Tübingen University, Tübingen, Germany. It was scanned by current voltage of 135 kV, intensity of the source current45 µA and voxel size resolution 6.29799 µm. The 3D model was built by segmenting the scans with the Amira® software. Teeth and inner ears were segmented manually. The cranial reconstruction was obtained using an automatic function of Amira® based on density detection. Additionally, in order to compare the inner ear of the Jradzor shrew with several extant eulipotyphlan taxa (five Soricidae, two Talpidae and one Erinaceidae) have been CT-scanned. They come from the Natural History Museum of Basel (NMB) and the collection of the University Claude Bernard Lyon 1 (UCBL). 3D inner ear models for all mentioned Eulipotyphla (excepted *Sorex monticolus*) were obtained with the GE Phoenix Nanotom CT-scanner from Basel operating at from 80 to 120 kV for comparison purposes. The intensity of the source current was set between 100 and 230 µA with voxel size resolution from 0.10 to 0.40 µm depending on the specimen (see Supplementary 1 for the detailed associated data).

## Institutional abbreviations

UCBL-FSL = University Claude Bernard Lyon 1 (Former Faculté des Sciences de Lyon); NMB = Natural History Museum Basel; IGS = Institute of Geological Sciences, National Academy of Sciences of the Republic of Armenia, Yerevan.

## Anatomical and measurements abbreviations

*Upper teeth terminology*: **I** = incisor, **A** = antemolar, **P** = premolar, **M** = molar.

*Teeth measurements*: **L** = total length, **W** = total width, **aW** = anterior width, **pW** = posterior width, **LT** = talon length, **bL** = buccal length, **lL** = lingual length, **peL** = posterior emargination length, **aW** = anterior width, **pW** = posterior width.

*Cranium terminology:* **afmi** = lower articular facet of the mandible, **afms** = upper articular facet of the mandible, **Bd** = bony bridge, **bs** = basioccipital suture, **Fbl** = foramen basisphenoidalis lateralis, **Fgp** = greater palatine foramen, **Fi** = incisive foramen, **Fio** = infraorbital foramen, **Fl** = lachrymal foramen, **Fo** = foramen ovale, **Fsi** = foramen supra infraorbitalis, **hp** = hamulus of pterygoid, **pb** = petrosal bone, **pf** = pterygoid fossa, **zp** = zygomatic processes.

*Inner ear terminology and measurements*: **[a, p, l]a** = [anterior, posterior, lateral] ampulla, **[a, p, l]sc** = [anterior, posterior, lateral] semi-circular canal, **ascL** = anterior semi-circular canal length, **ascW** = anterior semi-circular canal width, **aW** = anterior width, **ca** = cochlear aqueduct, **cc** = common crus, **ccL** = common crus length, **fc** = cochlear fenestra, **co** = cochlea, **co.ratio** = cochlea ratio (height divided by width), **es** = endolymphatic sac, **peL** = posterior emargination lenght, **pW** = posterior width, **ratio** = height divided by width, **sac** = saccule, **ut** = utricule, **va** = vestibular aqueduct, **fv** = vestibular fenestra, **fv.ratio** = vestibular fenestra ratio (height divided by width).

### Systematic paleontology

Family Soricidae Fischer, 1814

Subfamily Soricinae Fischer, 1814

Neomyini Matschie, 1909

Genus *Asoriculus* Kretzoi, (1959)

(type species: *Crocidura gibberodon* Petényi, 1864)

Original diagnosis of *Asoriculus* from Kretzoi (1959): “Compared to the known *Soriculus* and *Nesiotites* species, this species is characterised by primitively built lower I, deviating proportions of C and P, as well as smaller dimensions.” [Translated from German].

*Asoriculus gibberodon* (Petényi, 1864).

Figs 3-4

Emended diagnosis (upper teeth only) of *Episoriculus gibberodon* from Reumer (1984): “A rather small member of the Soriculini with only weakly pigmented teeth; four upper antemolars, of which the A4 may be variable in its development; upper molars variable in their morphology, with a moderate posterior emargination.”

Distribution: First appearance in Hungary during the MN12 (Tardosbánya: Mészáros, 1998). Presence in Spain, France, Austria, Germany, Poland, Romania, Slovakia, Hungary, Italy, Bulgaria, Greece, Turkey and Morocco until the mid-Pleistocene (Botka & Mészáros, 2017; Geraads, 1995; Reumer, 1998b; Rofes & Cuenca-Bescós, 2006; Rzebik-Kowalska, 1998).

#### Measurements (mm)

Left tooth row:** I**: L = 1.49, LT = 0.57, **A1**: L = 0.72, **A2**: L = 0.59, **A3**: L = 0.43, **A4**: L = 0.37, **P4**: bL = 1.65, lL = 1.05, peL = 0.97, W = 1.35, **M1**: bL = 1.54, lL = 1.38, peL = 1.20, aW = 1.46, pW = 1.63, **M2**: bL = 1.28, lL = 1.23, peL = 1.07, aW = 1.58, pW = 1.49, **M3**: L = 0.70, W = 1.22.

Right tooth row: **A3**: L = 0.38, **A4**: L = 0.36, **P4**: bL = 1.66, lL = 1.05, peL = 0.97, W = 1.38; **M1**: bL = 1.55, lL = 1.35, peL = 1.21, aW = 1.52, pW = 1.72, **M2**: bL = 1.27, lL = 1.22, peL = 1.12, aW = 1.59, pW = 1.52, **M3**: L = 0.67, W = 1.21.

**Description**

**Cranium**: The dorsal part the skull (Fig. 3A, B) is poorly preserved. Posteriorly, most of the squamosal, parietal and occipital bones are preserved but crushed into pieces so the contact between them is not visible. For the same reason the lambdoid crest, the occipital condyles and the sagittal crest are not preserved. Anteriorly, the rostrum is partially preserved but the premaxillary, maxillary and nasal bones are broken so their contacts are lost.

The buccal view of the specimen (Fig. 3D) confirms that most of the dorsal part of the skull is indeed crushed. The parietal foramen is consequently not observable. In contrast, the infraorbital and lachrymal foramen are well preserved. The infraorbital foramen is located above the P4 and the anterior part of the M1. The relatively wide lachrymal foramen is about half the diameter of the infraorbital foramen. It is located at the level of the infraorbital foramen and above the middle anterior part of the M1. The bony bridge separating both foramina is narrow. More anteriorly, above the A3, the supra infraorbitalis foramen is also preserved.

The basicramium is better preserved in occlusal view (Fig. 3C), so several anatomical features can be described. The left tooth row is fully preserved. It is composed of one incisor, four antemolars, one premolar and three molars. The size of the antemolars decreases from A1 to A4, whereas the P4 is much larger. The M1 is the largest molar, about as large as the P4, whereas the M3 is the smallest. Between the tooth rows, the palate is also well preserved. Both incisive foramen are located at the contact between the maxillar and premaxillar but mostly open in the premaxillary part of the palate. The greater palatine foramen is well preserved on the right side of the palate next to the M1 (visible but partly crushed on the left side). The posterior part of the maxillary shows a small zygomatic process ending before the level of M3 mesostyle.

The upper and lower articular facets of the mandible are preserved on both sides of the skull. The hamulus of the pterygoid is curved buccally and forms a loop enclosing the foramen ovale. The two basisphenoidalis lateralis foramina are visible on both sides, at the posterior end of the pterygoid fossa, where a fragment of the vomer is also preserved. The ectotympanic rings are not preserved, but both petrosal bones, located laterally on both sides of the basioccipital suture, are relatively close to the medial axis of the skull. Finally, the foramen magnum is visible at the posterior end of the skull, but the occipital area is heavily damaged, preventing further description of its anatomy.

**I:** I is strongly fissident and its main apex is hook-like. In lateral view, the talon is well developed, triangular and pointed. An undulated cingulum is present, ending as another small cusp in lingual view. In buccal view, an internal groove is opening posteriorly along the root.

**A1-A4:** These teeth overlap each other, and A3 but most notably A4 are compressed anteroposteriorly. In buccal view, all these upper antemolars have rather triangular shape, whereas their outline is rather quadrate in occlusal view. There are slightly recurved lingually. A1 and A2 are similar in height, A3 is slightly smaller, and A4 is less than half the size of A3. A weak cingulum surrounds the teeth but disappears anteriorly. In lingual side, the cingulum makes a small cusp aligned with the main cusp and linked to it by a faint crest. A faint cingulum conule is also present at the posteriolingual corner. In occlusal view, all are more stretched buccally than lingually. The A4 is fully visible in buccal view.

**P4:** The anterior border is oblique with the paracone being more anteriorly located than the protocone. The hypoconal and metaconal flanges are both strongly developed, delimiting a deep posterior emargination.

**Fig. 4:**
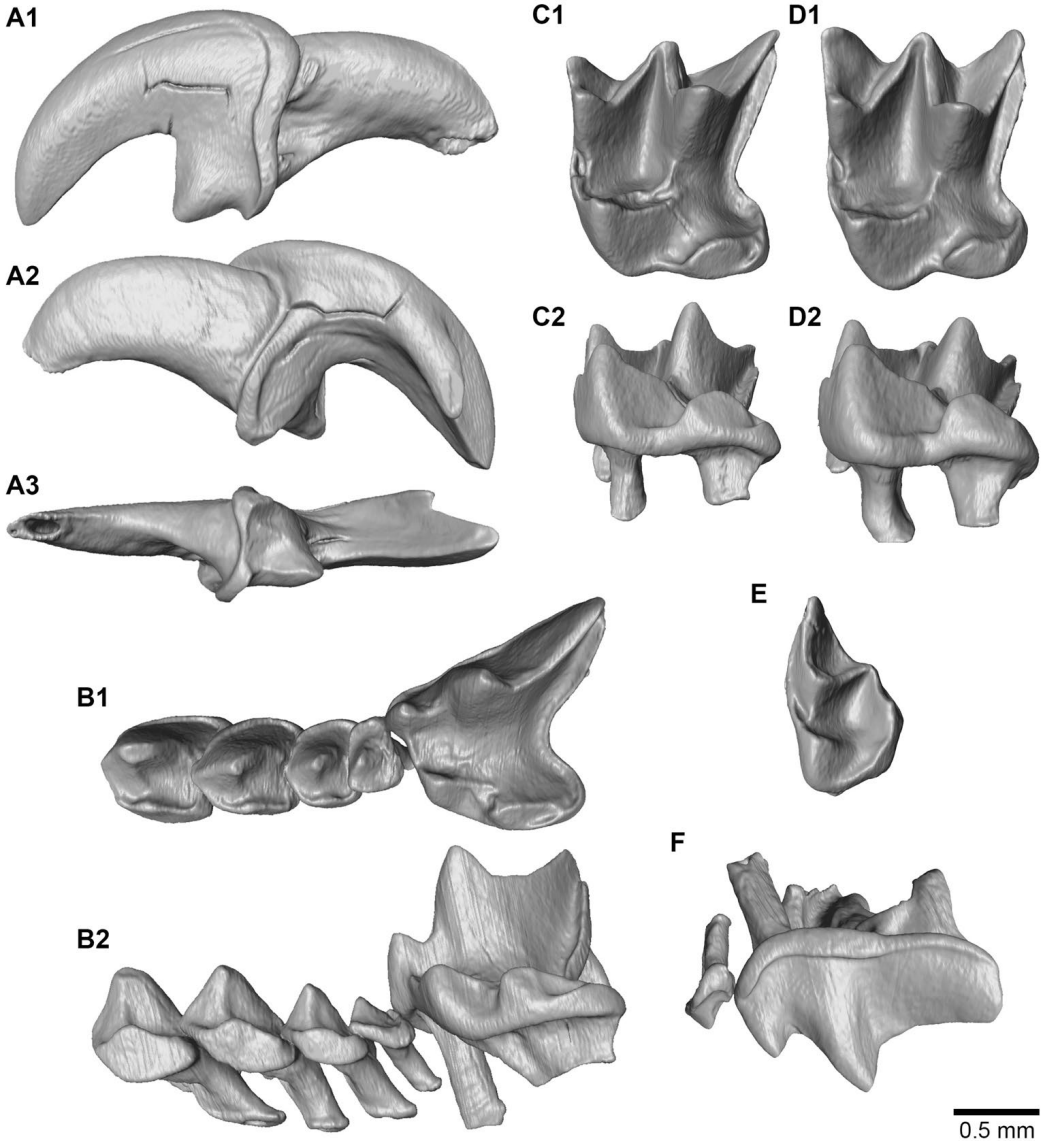
3D models of the teeth of *Asoriculus gibberodon* extracted from CT-data: **A** first left upper incisor (**A1**. buccal, **A2**. Lingual and **A3**. occlusal views); **B** left A1-P4 row (**B1**. Occlusal, **B2.** lingual view); **C** left M1 (**C1**. occlusal and **C2**. lingual views); **D** left M2 (**D1**. occlusal and **D2**. lingual views); **E** M3 in occlusal view; **F** A4 and P4 in buccal view

In buccal view, the P4 paracone is large and conical; its postparacrista is moderately long and has a constant height. In lingual view, protocone, hypocone, and the cingulum of the hypoconal flange have a stepped descending shape (Fig. 4. B2).

**M1-M2:** In occlusal view, the posterior emargination is less pronounced than that of the P4 (the hypoconal flange is short with no contact with the posterior tooth), and the anterior margin is not oblique. The protocone is U-shaped with a well-developed metaloph and a small anterior conule. The division of the paraloph forms a small pit on the anterior border between the paracone and the protocone. The metacone and the protocone are connected either by a prolongation of the postprotocrista (metaloph) or by an additional low crest that connects them directly. There is no hypoloph so the hypocone is isolated from the protocone by a deep sinus. The hypoconal flange is broad and rounded, and extends posteriorly further than the metastyle. The posthypocrista is short and curved toward the posterior emargination.

**M3:** It is half the length of M2. It shares the features of M1-M2 but lacks hypocone and metastyle. The posterior cingulum bears two faint conules, the lingual one being probably a vestigial metacone.

### Comparisons

Dental pigmentation, mandible shape, and lower teeth morphology are often used as key-characters for shrew identifications (e.g., Repenning, 1967; Reumer, 1998a; Zaitsev & Rzebik-Kowalska, 2004) that we cannot use for the Jradzor cranium identification. However, additional characters such as the fissident upper incisor (Repenning, 1967; Reumer, 1998a), the number and the relative size of antemolars (Hutterer, 2005b), the buccal shape of P4 (Repenning, 1967), the presence of metalophs and hypocones in upper molars (Repenning, 1967) allow to refer our specimen to the Soricinae subfamily and Neomyini tribe (Reumer, 1998a). We observed a well-pronounced buccal curvature of the hamulus at the base of the skull in the Jradzor specimen, a feature that is also strongly expressed in the Neomyini tribe but absent in other Soricinae, such as the genus *Sorex* (as noted by the authors).

Among the Neomyini tribe, five genera have four upper antemolars: *Nesiotites* Bate, 1945 (although the number of antemolars is variable between 3 and 4, see Reumer, 1980 and Pons-Monjo et al., 2012), *Neomys* Kaup, 1829, *Soriculus* Blyth, 1854, *Episoriculus* Ellermann & Morrison-Scott, 1966, and *Asoriculus* Bate, 1945 (Dubey et al., 2007; Repenning, 1967).

The cranium of Jradzor differs from *Neomys* by its generally smaller size and the shape of its antemolars: almost rectangular in occlusal view, main short cusp at the anterior part of the teeth and posteriorly recurved, lingual cusp poorly developed and posteriorly positioned. It also slightly differs from the *Soriculus* and *Episoriculus* by the position of its lachrymal foramen, anteriorly and dorsally displaced, compared to other Neomyini shrews (Francisk et al., 2019; Motokawa & Lin, 2005).

The taxonomy of *Asoriculus* is in need of a revision. Six species of *Asoriculus* have been described and are still considered valid so far: *A. burgioi* Masini & Sarà, 1998, *A. corsicanus* (Bate, 1945), *A. gibberodon* (Petényi, 1864), *A. similis* (Hensel, 1855), *A. maghrebiensis* Rzebik-Kowalska, 1988, *A. thenii* Malez and Rabeder, 1984 (e.g., Geraads, 1995; Janossy, 1973; Malez and Rabeder, 1984). These six species, with others, have been discussed to be either morphotypes, subspecies, or even juvenile ontogenetic stage of the same species *Asoriculus gibberodon* (e.g., Reumer, 1984; Rzebik-Kowalska, 1994). However, these points of views are not widely accepted and there is no consensus so far on the systematics of this genus. For example, Koufos et al. (2001) described seven morphotypes of *Asoriculus* in Osztramos 7; most recent studies also keep maintaining the use of several morphotypes or sub-species (e.g., Vasileiadou & Doukas, 2022). However, solving this systematic issue is beyond the scope of the present study.

Our specimen is close to the Reumer’s molars morphotype A of *A. gibberodon* also described in Popov (2003) and Vasileiadou et al. (2012). Nevertheless, it differs from it by its metalophs and paralophs division, a feature that has been rarely described for *Asoriculus*. For instance, *A. gibberodon* from the early Pleistocene of Greece presents divided metalophs but not divided paralophs (Koufos et al., 2001).

Therefore, not knowing the morphological variability of the population from Jradzor, we simply refer this cranium to *Asoriculus gibberodon*. This identification is coherent with mandibles of *Asoriculus gibberodon* found from the slightly younger stratigraphic layers (JZ-3, JZ-3s, JZ-13) of Jradzor section. These layers are included in the FA2 sedimentary unit composed of pyroclastic tails deposited between 3.98 and 4.1 Ma. The FA2 lays above the FA1 sedimentary unit—the diatomite where from the skull has been recovered (Lazarev et al., 2023).

**Inner ears of** *Asoriculus gibberodon* from Jradzor

Fig. 5

**Fig. 5.**
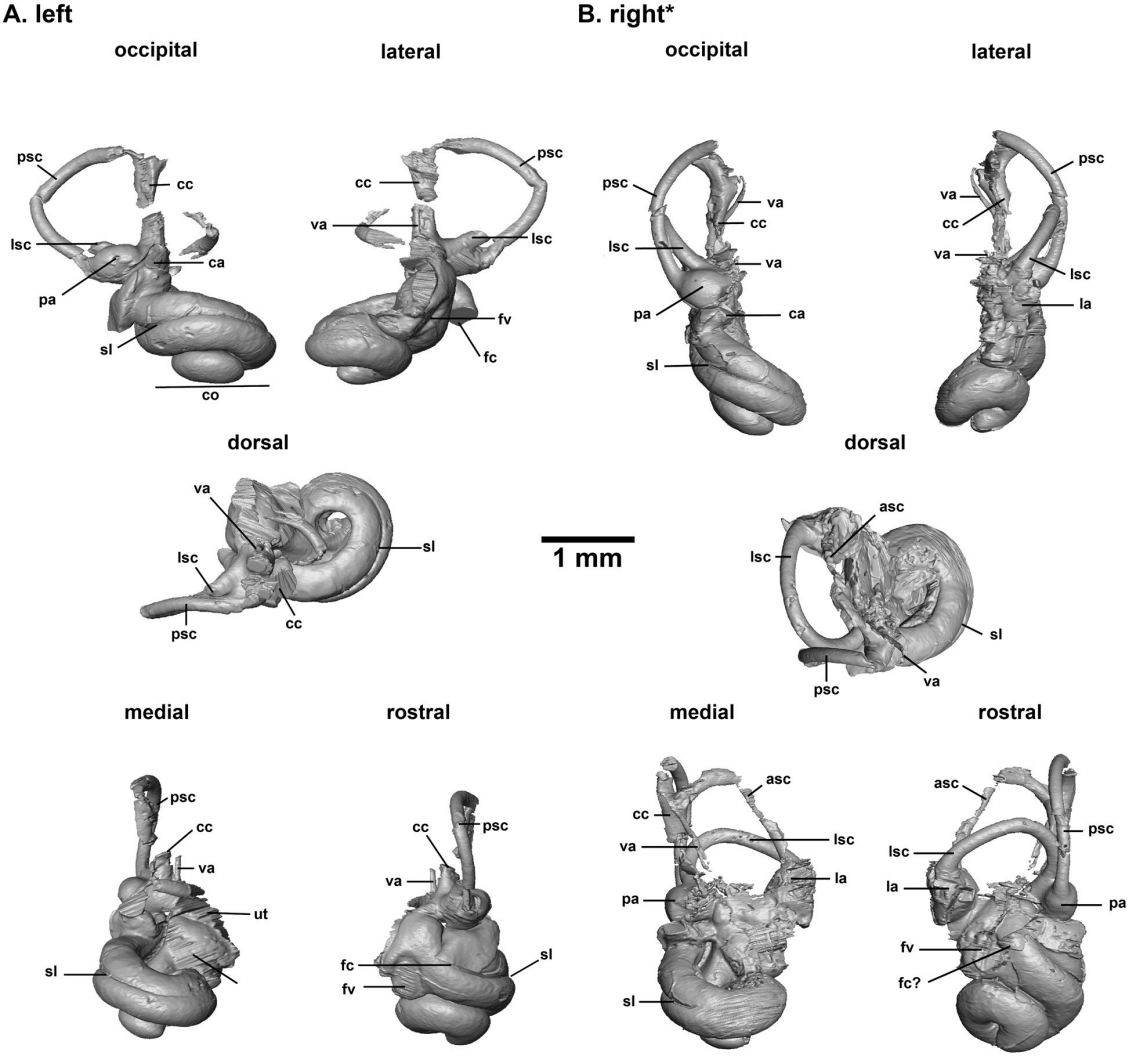
3D models of the inner ears of the Jradzor *Asoriculus gibberodon* cranium: **A** left labyrinth; **B** right labyrinth [*reversed illustration]; **[a, p, l]sc**: [anterior, posterior, lateral] semi-circular canal; **[a, p, l]a**: [anterior, posterior, lateral] ampulla; **cc**: common crus; **ut**: utricule, **sac**: saccule; **va**: vestibular aqueduct; **ca**: cochlear aqueduct; **fv**: vestibular fenestra; **fc**: cochlear fenestra; **co**: cochlea

**Description**

**Cochlea:** Both left and right cochleae are well preserved. The number of turns is about 1.5. A deep secondary bony lamina stretches over most of the basal turn. The blunt apex is detached from the basal turn. The latter is thick. The aspect ratio is low with a value of 0.48, and the cochlea is thus rather flat. The cochlear aqueduct could not be reconstructed. Its thick starting point on the right bony labyrinth seems to be medio-laterally oriented.

**Vestibule**: It is crushed on both bony labyrinths so that the saccule and utricule are not clearly visible. The stapedial fossa is well preserved on the left bony labyrinth. It is rather elliptical with a ratio of 1.55.

**Vestibular aqueduct:** It is poorly preserved on both bony labyrinths. Its starting point is visible on the left one and its course is apparently preserved on the right one. The aqueduct is thin and starts on the vestibule medially (more that the common crus). It is parallel to the common crus while being apparently slightly undulating. The aqueduct extends at least up to the splitting point of the common crus.

**Semi-circular canals:** The semi-circular canals are crushed or broken on both bony labyrinths and especially strongly flattened on the right one. However, it is possible to observe that the lateral semi-circular canal enters the vestibule posteriorly slightly in front of the posterior ampulla, preventing the formation of a secondary common crus. The posterior semi-circular canal has a slight undulation. The posterior and anterior semi-circular canals seem to extend beyond the dorsalmost extension of the common crus. The visible ampullae at the base of the semi-circular canals are rather ellipsoid in shape but still quite bulky.

**The bony labyrinth of extant eulipotyphlans**

Fig. 6-7

### Soricidae

**Fig. 6.**
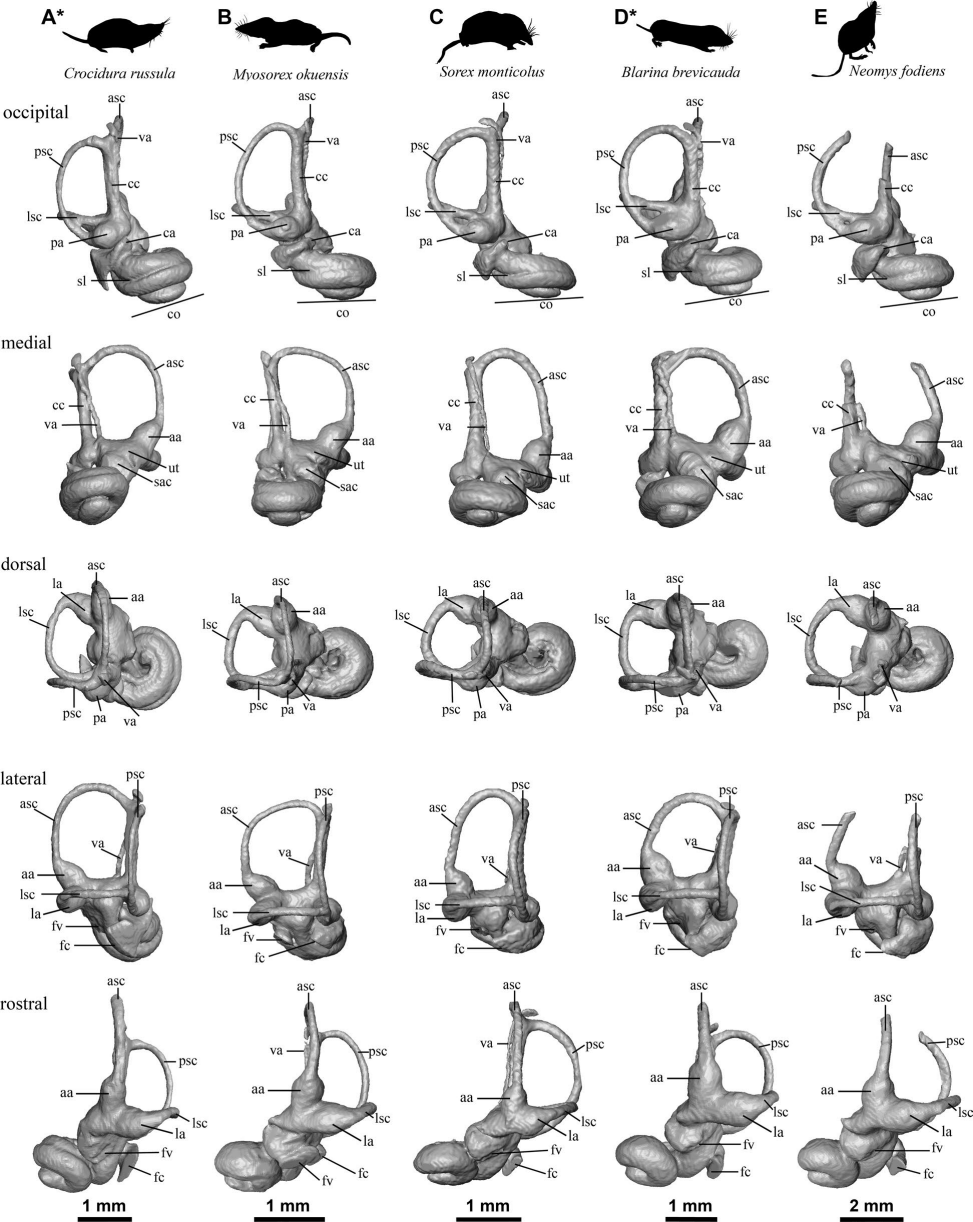
3D models of the inners ear of several extant species of Soricidae. Only left inner ears are illustrated except of marked by an asterisk (*), where mirrored right inner ear is illustrated: **A** Crocidurinae, *Crocidura russula* (NMB 9400); **B** Myosoricinae, *Myosorex okuensis* (UCBL-FSL 218024), **C** Soricinae, *Sorex monticolus* (www.digimorph.com); **D** Soricinae, *Blarina brevicauda* (NMB 4848), **E** Soricinae, *Neomys fodiens* (NMB 8697). See text for abbreviations

**Fig. 7.**
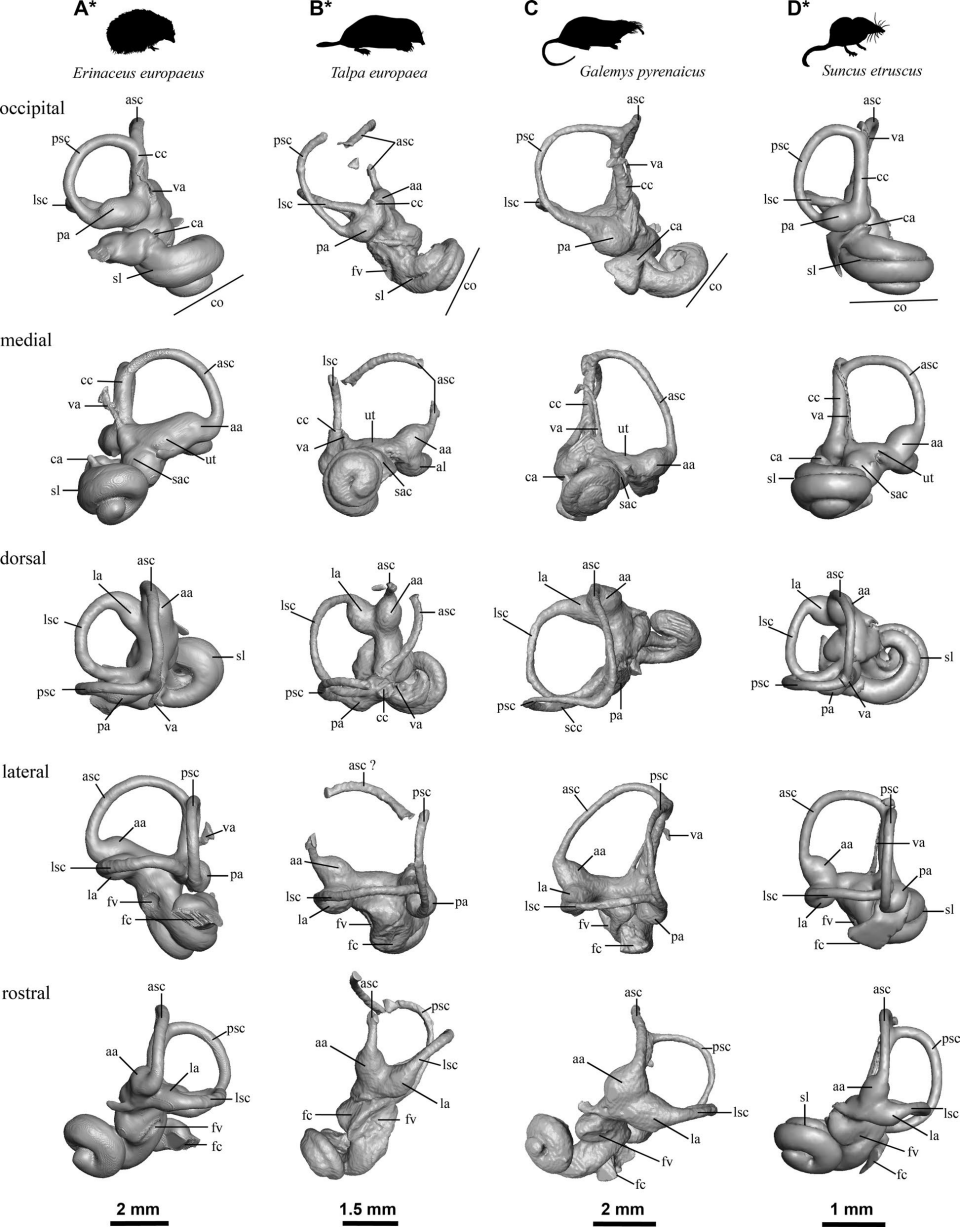
3D models of the inners ear of several species from different families of Eulipotyphla. Only left inner ears are illustrated except of marked by an asterisk (*), where mirrored right inner ear is illustrated: **A** Erinaceidae, Erinaceinae, *Erinaceus europaeus* (NMB 7008); **B** Talpidae, Talpinae, *Talpa europaea* (NMB 9757); **C** Talpidae, Talpinae, *Galemys pyrenaicus* (NMB 9415), **D** Soricidae, Crocidurinae, *Suncus etruscus* (NMB 6053). See text for abbreviations

Among the studied Soricidae, the proportions and general shape of the bony labyrinths do not differ significantly. Their general morphology corresponds to the description of the bony labyrinth of the *Sorex monticolus* specimen provided by Ekdale (2013). The common features for all soricid taxa are a cochlea with 1.5 turns; a bulbous basal turn; an ellipsoid fenestra vestibuli (with ratios between 1.4 and 2); a thick origin of the cochlear aqueduct with a similar mediolateral orientation; a higher than large posterior canal; a semi-lateral canal directly entering the vestibule posteriorly and positioned high relative to the posterior ampulla; and the origin of the vestibular aqueduct medial to the common crus. The endolymphatic sac is small in all Soricidae and usually extends just slightly above the end of the common crus, forming a small hook covering the latter part, except in *Myosorex okuensis* where it stays medial and reaches the top of the anterior semi-circular canal. The delicate nature of the skull bones of soricids and their small size explains the limited extent of the endolymphatic sac. A feature apparently common to the Crocidurinae is the persistence of the secondary bony lamina beyond the basal turn of the cochlea. This feature is slightly more variable within the studied Soricinae and Myosoricinae shrews and generally disappears after the first half to two-thirds of the basal turn (except for *Neomys fodiens*), but it is in general quite well marked. The angle between the basal turn of the cochlea and the common crus is slightly variable from one taxon to the other with a rather high angle approaching orthogonality, except in *Crocidura* (Figs. 6 and 7, occipital view). The canals are generally shorter and more robust in *Blarina brevicauda* and *Suncus etruscus*, while they are more elongated in the other taxa. The lateral canal is flattened at the top in all taxa except in *N. fodiens* and *Sorex monticolus*. The posterior canal is slightly more curved posteriorly in *B. brevicauda*, *M. okuensis*, and *Su. etruscus* extending its curvature above the most dorsal extension of the common crus. It is more circular in three Soricinae and more ellipsoid in the other taxa. The anterior semi-circular canal is flattened dorsally in *M. okuensis* and *Su. etruscus* giving it a slightly squarer shape.

### Erinaceidae and Talpidae

In Erinaceidae, the bony labyrinth of *Erinaceus europaeus* is more robust than that of our soricid sample. The semi-circular canals are short and thick and are more circular in shape. The angles between their planes are all almost orthogonal. The lateral canal enters the vestibule posteriorly at the level of the midline of the posterior ampulla. The vestibular aqueduct is also short, ending at half the length of the common crus. It is not parallel to the common crus and runs posteriorly from its origin in the vestibule and medially relative to the common crus. The fenestra vestibuli is elliptical with a ratio of 1.63. A faint secondary bony lamina runs over two-thirds of the massive basal cochlear turn. Both moles (*Talpa europaea* and *Galemys pyrenaicus*) show noticeably different morphologies. Compared to the Soricinae, moles have a significantly more coiled (two turns or more) cochlea that is also proportionally smaller relative to the size of the whole bony labyrinth. The cochlea is more detached from the vestibule than in *Erinaceus*. The vestibular aqueduct opens medially relative to the common crus, starting parallel to it but twisted posteriorly to open on the endolymphatic sac at about two-thirds of the common crus length in *G. pyrenaicus*. The semi-circular canals are thin and elongated, and do not extend beyond the common crus. Both posterior and lateral canals are flattened at their extremity, while the anterior canal is flattened obliquely. The angles formed between the planes of the semi-circular canals are generally orthogonal. The cochlea of *G. pyrenaicus* and *T. europaea* is flattened (aspect ratio above 0.9), with only a faint secondary bony lamina in *Galemys*, and the plane of the basal turn in the latter is twisted in comparison to all other studied Eulipotyphla. *T. europaea* has a slightly longer secondary bony lamina, deep in the first-third of the basal turn. Both moles show a tilted cochlea with regards to the vestibule or the common crus, a very different situation as in soricids (Fig. 7, occipital view). *G. pyrenaicus* has a lateral semi-circular canal entering the vestibule through the posterior ampulla, almost fusing with the posterior semi-circular canal and forming a secondary common crus with it. In contrast, *T. europaea* has a lateral canal entering quite high in the vestibule above (dorsally to) the posterior ampulla.

## Discussion

Only few soricid crania are known in the fossil record (e.g., Reumer, 1984; Storch & Qiu, 2004; Vanishvili, 2006). However, since post-palatal bones of the skull are very thin, and they are hardly ever preserved (Repenning, 1967), comparable finds are very rare. Consequently, most of the studied cranial parts are either fragments of maxillaries or nasal-palatal areas of the skull (e.g., Jin et al., 2009; Moya-Costa et al., 2019; Popov, 2003; Rofes & Cuence-Bescós, 2009). Additionally, due to the less diagnostic features of the upper dentition and the poorly known cranial anatomy of fossil soricids, more complete cranial remains have often been overlooked whenever available (e.g., Reumer, 1984; Storch & Qiu, 2004; Vanishvili, 2006). As a result, the knowledge on the phylogenetic relationship between fossil Soricidae and extant species is mainly based on the dental and mandibular anatomies (e.g., Hugueney & Maridet, 2011; Rofes & Cuenca-Bescós, 2009), and recently with new methodologies coupling molecular and morphological data (Yuan et al., 2024). In contrast, the cranial anatomy of extant species and its phylogenetic implications are often studied (e.g., Carraway, 2010; Esselstyn et al., 2021; Jenkins et al., 2009, 2013; Konečný et al., 2020; Maier et al., 2022), on top of the already well-known phylogenetic relationships based on molecular data (e.g., Bover et al., 2018; Douady et al., 2002; Dubey et al., 2007; Fumagalli et al., 1999; Ohdachi et al., 2005; Quérouil et al., 2001).

The genus *Asoriculus* was erected by Kretzoi (1959) based on a few morphological differences compared to the genera *Soriculus* and *Nesiotites*, thus already suggesting a close phylogenetic affinity with them. During the Pliocene, the genus is distributed in all the peri-Mediterranean area but does not occur in the Circum-Paratethyan realm (Botka & Mészáros, 2017; Furió & Angelone, 2010; Geraads, 1995; Reumer, 1998b; Rofes & Cuenca-Bescós, 2006; Rzebik-Kowalska, 1998). The Armenian record represents its most eastern occurrence.

Since Hutterer (1994), several species of Pliocene and Pleistocene shrews referred to *Episoriculus* are now referred to *Asoriculus*. Hutterer (1994) stated that *Soriculus*, *Episoriculus* and *Chodsigoa* are not closely related to each other, thus suggesting a closer affinity of *Asoriculus* with *Episoriculus*. More recent molecular phylogenetic analyses have further investigated the relationships between the genera usually referred to the Nectogalini tribe, equivalent to Neomyini herein. Dubey et al. (2007) confirmed that *Episoriculus*, *Chodsigoa*, *Chimarogale* and *Neomys* form a monophyletic clade corresponding to the Nectogalini tribe. Later, Liu et al. (2017) showed an overall close affinity between *Episoriculus*, *Soriculus* and *Nectogale,* whereas Bover et al. (2018) found that *Soriculus* and *Nesiotites* are closer to each other and *Episoriculus* and *Chodsigoa* form together a sister clade. Rofes and Cuenca-Bescós (2009) provide so far the only phylogenetic analysis including the genus *Asoriculus* based on teeth and mandible morphologies. Their study suggests that *Asoriculus gibberodon* is closer to *Nesiotites ponsi* and *Nesiotites hidalgo* than to *Soriculus*, thus proposing that *Asoriculus* could be an ancestor of *Nesiotites*. However, the genus *Episoriculus*, which is polyphyletic (He et al., 2010), was not included in their analysis.

Among Neomyini, as far as the cranium anatomy is known, the specimen from Jradzor is most similar to that of *Soriculus* and *Episoriculus*, with only a slightly more anterior and dorsal position of the lachrymal foramen. Our cranial anatomic observations alone consequently point to a closer affinity of *Asoriculus* with *Soriculus* and *Episoriculus* among extant Neomyini. However, most of the cranium morphology of *Nesiotites* remains unknown. Thus, it is impossible to test further the hypothesis of *Asoriculus* being an ancestor to *Nesiotites*.

Compared to a sample of extant soricids, talpids and an erinaceid, the bony labyrinth of *A. gibberodon* from Jradzor also resembles that of a typical soricid. It has an ellipsoid posterior canal, a medial origin of its vestibular aqueduct, a small endolymphatic sac overlying the dorsal-most extent of the common crus, a point of entry of the lateral semi-circular canal high on the dorsal side of the posterior ampulla in soricids (very different in *Talpa, Galemys* or *Erinaceus;* Fig. 7), a very similar general cochlear shape, a short cochlea (1.5 turns) with a visible secondary bony lamina in general (and even very marked in *Asoriculus*, see below for further discussion), a bulbous basal turn. *Asoriculus* fits perfectly the soricid anatomic pattern, confirming the strong association between phylogenetic affinities and the morphology of the bony labyrinth (e.g., Ekdale, 2013; Mennecart et al., 2022; Urciuoli et al., 2021). Beyond these observations, it is impossible in this study to use the bony labyrinth of *A. gibberodon* to understand its phylogenetic relationships with other soricids better. Further morphometric and statistical methods and analysis are hampered by the deformation and breakage of the studied specimen. A significantly larger comparative dataset of extant taxa will be also required, which, again, is beyond the scope of this study.

### Auditory capacities

Auditory capacities in shrews have been extensively studied since Gould et al. (1964) and Buchler (1976) who experimentally found out that they could produce high-frequency calls and clicks. Rare preservation of the bony labyrinth in a fossil species such as here with *A. gibberodon* can give insights into hearing capacities and evolution of specific sensory abilities in the clade. To date, it is not known when shrews began to develop this ability during their evolution. Several extant species of the genera *Sorex*, *Blarina*, or *Crocidura* can indeed generate ultrasounds and even orient themselves, i.e. “echo-based orientation” (*sensu* Sanchez et al., 2019), using high frequencies of at least up to 110 kHz (Forsman & Malmquist, 1988; Thomas & Jalili, 2004), making them some of the few echo-based orienting terrestrial mammals. This ability in terrestrial animals seems to be related to nocturnal habits or life in low-light environments and as a means of sensing the environment, especially when crossing open areas (see Thomas & Jalili, 2004). Toothed whales and bats are true echolocating mammals, but they use it in the water and air respectively. Producing high-frequency sounds means that the animals can hear the echo produced when sounds bounce back against obstacles. It requires a specific cochlea and hair cell arrangement within the latter. In dolphins and bats, a very stiff basilar membrane (Ekdale, 2013; Fleischer, 1976) runs along the midline of the cochlea and moves according to the frequency of the incoming sounds. This structure, present in all mammals but particularly stiff in echolocating taxa, leaves a deep groove on the outer surface of the cochlea, and, thus, on the bony labyrinth reconstructed from the endocast of the cochlea. It is known as secondary bony lamina. While it is extremely visible in bats and dolphins (e.g., Costeur et al., 2018; Davies et al., 2013a, 2013b), it is not as evident in the bony labyrinth of shrews (see Figs. 6 and 7), attesting to their ability not to echolocate but rather only to orient themselves based on simple high-frequency calls and clicks. Echo-based orientation behavior has been observed in present-day shrews when they are in unfamiliar environments. This capability is essential for organisms with high metabolic rates, allowing them to quickly map their surroundings without much energy expenditure, and it is, therefore, frequently utilized by wandering shrews (Tomasi, 1979). The secondary bony lamina of the Etruscan shrew (*Suncus etruscus*) is nevertheless particularly marked and long compared to shrews for which it is known, probably indicating a good high-frequency auditory adaptation. However, the secondary bony lamina is very subtle in the wandering shrew *Blarina* which regularly uses ultrasound for orientation (Fig. 6) (Tomasi, 1979), indicating that the absence of a deep secondary bony lamina doesn’t necessarily contradicts an auditory sensitivity related to echo-based orientation in shrews. Despite contradictory preliminary analyses stating that the animal had poor hearing capacities, shrews rely much more on tactile stimuli to sense their environment (Berg, 2016). Short cochleae with loose turns are also usually found in taxa specialized in high frequencies because high-frequency sounds do not travel along long distances (Forsman & Malmquist, 1988; Ketten, 1992; Tomasi, 1979). Comparison of the secondary bony lamina among Soricidae, Talpidae and Erinaceidae indicate that most of soricids have a long and well-marked lamina, particularly well visible on *Suncus*, *Crocidura*, *Sorex*, *Neomys* (Figs. 6 and 7) and also *Asoriculus* (Fig. 5) from Jradzor. Likewise, the overall short length of the cochlea and the loose coiling pattern in all soricids, including *Asoriculus,* also point to a specialisation towards high frequencies with a noticeable deep secondary bony lamina and a very soricid-like general cochlear shape. No morphological difference can be found between semi-aquatic or fully terrestrial shrews (e.g., *Neomys* vs. *Crocidura*), thus, confirming that high-frequency adaptation in shrews is not a main system of orientation (Catania et al., 2008). We tentatively infer a good high-frequency auditory capacity for the fossil *Asoriculus*, and an echo-based orientation system in this taxon is consequently likely but cannot be confirmed yet.

### Locomotion mode and agility

Soricids show a range of ecological adaptations between fully terrestrial, arboreal and semi-aquatic habits (Hutterer, 1985). Such diversity requires a range of locomotion modes and abilities. *Asoriculus* is mostly known from fossil teeth, and thus, little is known about its locomotor adaptations. The fossil from Jradzor is a relatively complete skull with good preservation of the ear region, a very rare case for fossil soricids. The ear region houses the vestibular system responsible for the sense of balance and spatial orientation. The semi-circular canals of the vestibular system are filled with endolymph, a fluid that moves when the head moves and stimulates hair cells situated within the ampullae at their base (Fife, 2010). Great attention has been given to the very variable shape and size of the canals in vertebrates because different ways of life involve very different abilities to sense the three-dimensional environment (e.g., Davies et al., 2013a, 2013b; Pfaff et al., 2015; Spoor et al., 2002, 2007), i.e. flying or moving fast in trees might indeed require more sensibility than spending most of its time in burrows, requiring different canal shapes to accelerate the fluids differently. This assumption led Spoor et al (2002) to develop a relationship between canal size and agility in mammals, where canals would be longer and thinner in very agile mammals. Intuitively, the stouter canals of the neither fast nor particularly agile hedgehog *Erinaceus* and the thinner canals of shrews (Figs. 6 & 7) seem to confirm this general rule. Interestingly, the semi-circular canals of *Atelerix*, the African spiny hedgehog, are longer and thinner than in its European counterpart for the same way of life (Ekdale, 2013). Shape variation of the semi-circular canals in archosaurs was recently shown to be statistically associated to locomotor preferences (such as bipedalism or flight; Bronzati et al., 2021). However, the authors relate this to geometric constraints in the braincase as well as to deep evolutionary divergences on the phylogenetic tree rather than to locomotor abilities. Large shape variation within bats and whales (toothed vs. baleen), from reduced to very reduced canals, or long and thin to small and thick canals (see Costeur et al., 2018 and Davies et al., 2013a, 2013b for examples), and even intraspecific shape variation related to relaxed evolutionary pressures (Billet et al., 2012; Mason et al., 2016) altogether show a more complex picture. The link between canal shape and size with locomotor abilities is thus far from being clear. Interpretations of speed capacities (Grohé et al., 2018) or underground adaptations (Mason et al., 2016) must be taken with caution. The clear shape difference between soricids and other eulipotyphlans studied here may thus be related to their phylogenetic distance and not to their locomotor abilities. The canals of *Asoriculus* are partly broken and displaced but they do not appear to show any shortening or thinness that could suggest a specific adaptation to 3D orientation or speed. Additionally, the occipital view in Fig. 5B seem to indicate a soricid pattern with a slight dorsal flattening. This morphology is very similar to that of *Blarina brevicauda*, *Myosorex okuensis,* and *Suncus etruscus*, but differs noticeably from the larger and rounder shape of *Neomys fodiens* and *Galemys pyrenaicus.* Although this morphology cannot be directly interpreted in terms of locomotion, it also suggests that *A. gibberodon*’s locomotion is closer to that of *Blarina brevicauda*, *Myosorex okuensis,* and *Suncus etruscus*, which could exclude a semi-aquatic adaptation. A 3D geometric morphometrics analysis is beyond the scope of this study, but including more taxa could maybe help identify potential ecological or geometrical constraints on the morphology of the bony labyrinth of Eulipotyphlans.

### Ecology of* A. gibberodon*

*Asoriculus gibberodon* has been interpreted by several previous studies (e.g., Botka & Mészáros, 2017; Furió et al., 2018; Maul & Rzebik-Kowalska, 1998; Reumer, 1984; Rzebik-Kowalska, 1995; Vasileiadou et al., 2003) as a terrestrial wandering shrew living in wet, closed or forested environment. Additionally, Furió (2007) suggested that the frequent occurrence of *Asoriculus* in the lacustrine sites from Orce (Fuente Nueva 3 and Barranco León) confirms the affinity of the genus with aquatic environments. However, Popov (2003) contradicts these interpretations and rather indicates warm and dry environments with a mosaic of open landscapes for the species. So far, any climatic reconstructions for the early Pliocene of the region is missing but geochemical data from the neighbouring region in Western Iran seem to further confirm this warm and dry interpretation of the climate (Böhme et al., 2021). However, the thick diatomite deposits in Jradzor mine attest to the existence of a large lake that was likely to sustain both more humid conditions and a surrounding forest. This confirms most of the previously proposed ecological requirements of *A. gibberodon* as a species living in rather wet environments and associated with permanent bodies of water, even if the regional climate and the surrounding landscapes are likely warm and dry. Furthermore, as previously discussed, the short length of the cochlea and deep secondary bony lamina indicate a specialisation towards hearing high frequencies for *A. gibberodon*, that is shared with present-day shrews, suggesting this character was already present in shrews 4 My ago. This specialization may suggest an echo-based orientation for *A. gibberodon*, which is also well known as an adaptation to assist shrews to move in their nearby surroundings especially to detect small insects or scramble about through grass and undergrowth when they cannot rely on other senses (e.g., Brinkløv et al., 2022; Siemers et al., 2009). An echo-based orientation that reduces energy expenditure in wandering and foraging would also be consistent with the characteristics of a terrestrial, humid environment and the vegetation surrounding permanent water bodies.

Although it is well known that terrestrial mammals can be fossilized in lacustrine deposits (e.g., Iskandar, 1990; Storch, 2001), the discovery of this cranium of *A. gibberodon* inside the diatomite, an environment corresponding to distal lacustrine settings, raises the question of the possible semi-aquatic adaptation of this species, as this adaptation is known for other species of the family. Indeed, semi-aquatic adaptations are known for four genera of extant shrews: *Sorex* (with the American water shrew *Sorex palustris* Richardson, 1828 and the Bendire's water shrew *Sorex bendirii* Merriam, 1884) and all the species of *Neomys*, *Chimarrogale* and *Nectogale*. As discussed above the bony labyrinth anatomy does not show any feature that can be related to semi-aquatic adapted life (by comparison with *N. fodiens* and *G. pyrenaicus*), and the slight differences observed on the inner ear among soricids rather point to a strictly terrestrial locomotion, although no definitive interpretation can be given to this point. Moreover, *Soriculus* and *Episoriculus*, being two genera closest to *Asoriculus* based on cranial anatomy, are not semi-aquatic, even if *Episoriculus* favors living in rather wet environments such as damp forests (Reumer, 1984; Walker, 1964).

The remain of a lagomorph specimen in association with the shrew suggests that terrestrial species could exceptionally be deposited and preserved in the lake (Lazarev et al., 2023). We thus propose that the lacustrine deposit responsible for the preservation of the lagomorph and the shrews does not represent their living environment but rather the depositional environment following *post-mortem* transportation.

## Conclusion

We described an exceptionally preserved skull of a specimen of *Asoriculus gibberodon* from the Pliocene of Jradzor, Armenia, and we compared its inner ears with those of present-day Eulipotyphla in order to make phylogenetic and ecological inferences.

The following results are underlined:

- We have assigned the Jradzor shrew to the species *Asoriculus gibberodon* of the Neomyini tribe based on the following synapomorphies: the fissident upper incisor, the presence of the 4th antemolar with a variable development, the buccal shape of P4, the presence of lophs on the molars, the buccally-curved hamulus and the position of its lachrymal foramen. Additionally, the Jradzor shrew exhibits a unique character that is the division of its lophs on its upper molars, which is interpreted as an intraspecific variability of *Asoriculus gibberodon*.
- The Jradzor shrew inner ears resemble those of a typical soricid: ellipsoid semi-circular canals, a 1.5 turn bulbous cochlea with a secondary bony lamina, a short endolymphatic sac overlying the dorsal-most extent of the common crus, a point of entry of the lateral semi-circular canal high on the dorsal side of the posterior ampulla. The preservation state of the fossil and the lack of data from extant species do not allow for a better understanding of the phylogenetic relationships of soricids.
- The shape of the cochlea and the presence of the secondary bony lamina indicate that the Jradzor shrew had good hearing capacities. However, while an echo-based orientation system is plausible, it cannot yet be confirmed. The morphology of the semicircular canals, which has been used to infer lifestyle in other clades previously, provides yet an unclear signal in soricids.
- A terrestrial lifestyle is mainly supported by the cranium anatomy of the Jradzor shrew that resembles that of terrestrial shrew, which is consistent with the previously proposed paleoecological model. We conclude that the *Asoriculus gibberodon* specimen from Jradzor inhabited wet or humid environments in close proximity to the lake under a regional dry climate, and was *post-mortem* deposited in the lake.

## Supplementary Information

Supplementary Material 1.

## Data Availability

No datasets were generated or analysed during the current study.
